# Dynamics of Rhizosphere Microbial Communities and Bioactive Compound Accumulation in Wild-Simulated *Astragalus mongholicus* Across Different Cultivation Years

**DOI:** 10.3390/plants15213493

**Published:** 2026-11-09

**Authors:** Ouli Xiao, Bin Ma, Xiaofeng Dai, Jieyin Chen, Zhiqiang Kong

**Affiliations:** 1 State Key Laboratory for Biology of Plant Diseases and Insect Pests, Institute of Plant Protection, Chinese Academy of Agricultural Sciences, Beijing 100193, China; xiaoouli123@163.com (O.X.); daixiaofeng_caas@126.com (X.D.); 2 Institute of Forestry and Grassland Ecology, Ningxia Academy of Agricultural and Forestry Sciences, Yinchuan 750002, China; mbin89@163.com; 3 Western Agricultural Research Center, Chinese Academy of Agricultural Sciences, Changji 831100, China

**Keywords:** *Astragalus mongholicus*, rhizosphere microbiome, wild-simulated cultivation, soil physicochemical characteristics, bioactive compounds

## Abstract

Knowledge regarding the structure and distribution patterns of rhizosphere microbial communities of *Astragalus mongholicus* (*A. mongholicus*) under wild-simulated cultivation remains limited. This study investigated how cultivation duration affects soil properties, microbial communities, and bioactive compound accumulation, as well as the relationships among these factors. High-throughput sequencing of the 16S rRNA and ITS regions was performed to profile bacterial and fungal communities in rhizocompartments of 1–4-year-old *A. mongholicus*. Meanwhile, soil physicochemical properties and bioactive compounds were quantified. Fungal community diversity in the rhizosphere of *A. mongholicus* varied across cultivation years, whereas bacterial community diversity showed a comparatively weaker response. The relative abundances of the potentially beneficial taxa Mortierellomycota and *Mortierella* in rhizosphere soil gradually declined with increasing cultivation age. Soil nutrient-related variables (OM, OC, P, and NH_4_^+^-N) were the main drivers shaping microbial community composition. Bioactive compound contents also fluctuated across cultivation years. Microbial genera including *Mortierella*, *Pseudomonas*, *Bacillus*, and the *Allorhizobium–Neorhizobium–Pararhizobium–Rhizobium* clade may be closely associated with the accumulation of bioactive compounds in *A. mongholicus*. These findings suggest that cultivation duration reshaped rhizosphere communities and bioactive compound accumulation in *A. mongholicus*. Soil nutrient availability and beneficial microbes were closely associated with these changes, providing insights for microbiome-informed quality improvement.

## 1. Introduction

The rhizosphere microbiota mediates interactions between plant roots and the soil environment, acts as a critical barrier against soil-borne diseases, and plays essential roles in plant growth, nutrient cycling, and soil health [1,2]. The rhizosphere is the soil zone most directly and intensely influenced by root activities and metabolism. Plants can selectively recruit specific microbial taxa by modulating the types and components of root secretions, thereby promoting the establishment of rhizosphere microbial communities and maintaining their dynamic equilibrium [3,4]. Therefore, the rhizosphere microbial community is not only a crucial component in plant adaptation to soil environments, but may also participate in processes including plant growth and quality formation [2,5]. Plant species, developmental stage, soil environment characteristics, cultivation practices, and climate affect rhizosphere microbial community composition [6,7,8,9,10]. Specifically, prolonged plant growth can continuously reshape root exudate inputs, soil nutrient status, and microbial habitats, thereby driving the dynamic succession of rhizosphere microbial communities. Consequently, the dominant rhizosphere microbial taxa and their abundances generally shift with planting duration [11,12,13]. For example, as cultivation duration increases, rhizosphere microbial diversity in *Panax ginseng* and *Panax quinquefolius* L. decreases, accompanied by a significant reduction in soil pH [14,15]. Li et al. [16] reported that the bacterial community structure of *A. mongholicus* cultivated for different numbers of years exhibited a time-decay pattern. These studies indicate that cultivation duration may serve as an important factor shaping rhizosphere microbial community succession in medicinal plants, whereas its underlying mechanisms may vary with plant species, cultivation patterns, and soil environments [17,18,19,20].

Beyond affecting soil ecological processes, rhizosphere microorganisms may also influence medicinal plant growth and quality through pathways such as nutrient transformation, plant growth regulation, and secondary metabolic modulation [21,22]. Environmental factors such as light, temperature, and soil conditions regulate the growth and development of medicinal plants, with the soil environment being particularly important [14,23]. Unlike traditional flatland cultivation, wild-simulated systems employ low-intervention agronomic management that mimics natural habitats [24]. Different cultivation practices can alter soil nutrient status, root exudate composition, and rhizosphere microbial communities, thereby potentially influencing the biosynthesis and accumulation of bioactive compounds in medicinal plants [20]. Rhizosphere microorganisms are linked to the secondary metabolism of medicinal plants. Specifically, astragaloside content was positively associated with the relative abundance of *Stenotrophomonas* in the rhizosphere, whereas the abundances of *Phyllobacterium* and *Inquilinus* in the roots were positively associated with calycosin content [16].

*Astragalus* is the dried root of *Astragalus membranaceus* (Fisch.) Bge. var. *mongholicus* (Bge.) Hsiao or *Astragalus membranaceus* (Fisch.) Bge., both of which belong to the family Fabaceae [25]. The major chemical constituents of medicinal *Astragalus* include flavonoids, saponins, and polysaccharides. It exhibits a broad range of pharmacological activities, such as anti-infective, cytoprotective, anti-inflammatory, antioxidant, antitumor, antidiabetic, and immune-enhancing properties [26]. The content of bioactive compounds varies among different *Astragalus* species and medicinal parts: dihydroflavones, isoflavones, and flavones are enriched in the aerial organs; isoflavans and saponins predominantly accumulate in the roots [27]. Liu et al. [28] detected 13 flavonoid and triterpenoid metabolites across different organs and developmental stages of *A. mongholicus* and *A. membranaceus* and found that calycosin-7-glucoside accumulates mainly in the roots. This suggests that cultivation duration and accompanying changes in soil environment and root-associated microorganisms may influence the accumulation of active components in the roots of *A. mongholicus*. To date, few studies have investigated the structure and distribution patterns of root-associated microbial communities in *A. mongholicus* transplanted to mountainous areas for wild-simulated cultivation. Therefore, this study focused on the medicinal plant *A. mongholicus*. We used high-throughput sequencing to characterize the composition of root-associated microbial communities in mountain-transplanted *A. mongholicus* across different cultivation years and UPLC–MS/MS to determine the contents of 35 bioactive compounds. This panel includes pharmacopoeia markers and encompasses a wide range of astragalosides, flavonoids, and soyasaponins.

In this study, we aimed to address the following questions: (1) Do rhizosphere bacterial and fungal communities exhibit differential sensitivities to cultivation years under wild-simulated cultivation? (2) Which soil physicochemical factors are the primary drivers of community shifts? (3) Are shifts in microbial community composition associated with changes in bioactive compound accumulation in roots?

## 2. Results

### 2.1. Rhizosphere Microbial Community Diversity of A. mongholicus Across Cultivation Years

**Figure 1:**
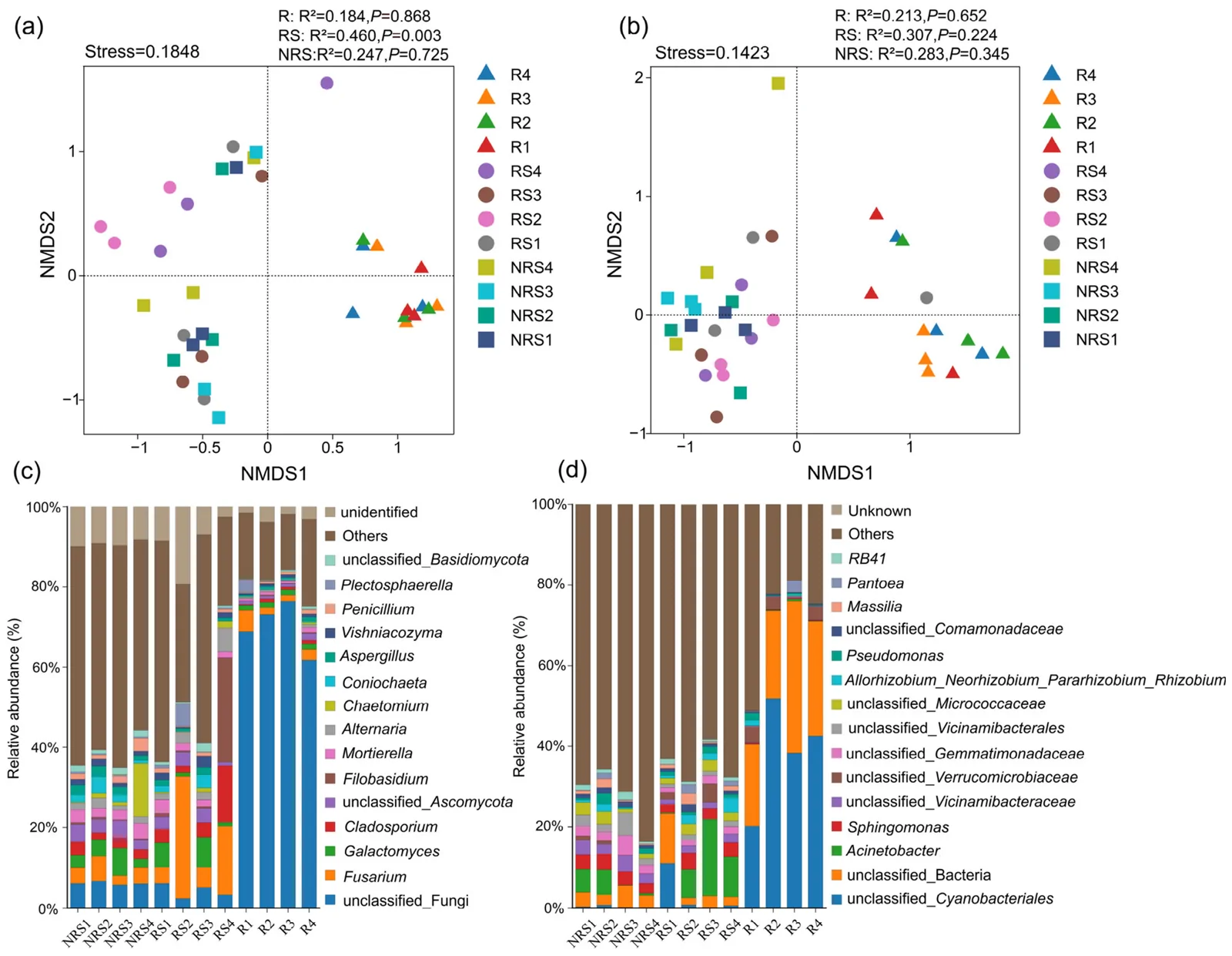
Nonmetric multidimensional scaling analysis (NMDS) of fungal (**a**) and bacterial (**b**) communities in non-rhizosphere soil (NRS), rhizosphere soil (RS), and roots (R) of *A. mongholicus* across different cultivation years. Genus-level species composition and relative abundance of fungal (**c**) and bacterial (**d**) communities.

Differences in the diversity of rhizosphere microbial communities of *A. mongholicus* across cultivation years were evaluated by comparing α- and β-diversity. Across cultivation years, the Shannon index of fungal communities was significantly lower in third-year non-rhizosphere soil (NRS3) (Figure S1). In contrast, the Shannon indices of fungal communities in first- and third-year rhizosphere soils (RS1 and RS3) were markedly greater than those in second-year rhizosphere soil (RS2), while the root sample from the fourth year (R4) exhibited the highest Shannon index. Neither the Shannon nor Chao1 index of bacterial communities showed statistically significant differences among cultivation years. The Shannon index of root-associated bacterial communities exhibited a decreasing trend as cultivation years increased, with the highest value observed in R1 (Figure S2). NMDS based on Bray–Curtis distances was implemented to assess the overall structures of fungal and bacterial communities associated with *A. mongholicus* across different cultivation years (Figure 1a,b). The results showed clear separation between root and soil samples; the rhizosphere bacterial communities did not show clear separation among the different cultivation years. These findings suggest that cultivation duration primarily influenced the diversity and community structure of fungi, whereas bacterial communities were comparatively less responsive, with sample type exerting a more pronounced effect on the overall microbial community composition.

### 2.2. Rhizosphere Microbial Community Composition of A. mongholicus Across Cultivation Years

The dominant fungal phyla in roots were Ascomycota, Basidiomycota, and Chytridiomycota (Figure S3a). The dominant fungal phyla in non-rhizosphere and rhizosphere soils mainly included Ascomycota, Basidiomycota, and Mortierellomycota. Notably, the relative abundance of Mortierellomycota in rhizosphere soil gradually declined as the cultivation period increased. The relative abundances were 3.52%, 1.92%, 1.71%, and 1.51%, respectively. At the bacterial phylum level, Cyanobacteria, Firmicutes, and Proteobacteria were the dominant phyla in root samples. In non-rhizosphere and rhizosphere soils, Proteobacteria, Actinobacteria, and Acidobacteriota were the dominant bacterial phyla. The relative abundance of Proteobacteria initially increased and subsequently decreased, reaching its highest level in NRS2 and RS2 (46.28% and 49.12%) (Figure S3b). At the genus level (Figure 1c,d), the relative abundances of *Fusarium*, *Galactomyces*, and *Cladosporium* differed markedly among rhizosphere soils from *A. mongholicus* cultivated for different durations. *Fusarium* was relatively abundant in RS2 and RS4 (30.47% and 17.09%), whereas *Galactomyces* accounted for a greater proportion in RS1 and RS3 (6.20% and 7.61%). In addition, *Alternaria*, *Cladosporium*, and *Filobasidium* exhibited their highest relative abundances in RS4. In R1, *Fusarium* and *Plectosphaerella* were relatively abundant. The bacterial community composition at the genus level in RS1 and R1 differed markedly from that in samples from other cultivation years. *Allorhizobium–Neorhizobium–Pararhizobium–Rhizobium* and *Pseudomonas* showed relatively high abundances in R1 (1.41% and 1.82%), whereas *Pantoea* was more abundant in R3 (2.89%). The progressive decline in Mortierellomycota and the year-specific enrichment of genera such as *Fusarium*, *Galactomyces*, *Cladosporium*, *Pseudomonas*, and *Pantoea* suggest that cultivation duration may drive taxonomic turnover in the root-associated microbiome.

### 2.3. Structural and Functional Variations in Microbial Communities in A. mongholicus

**Figure 2:**
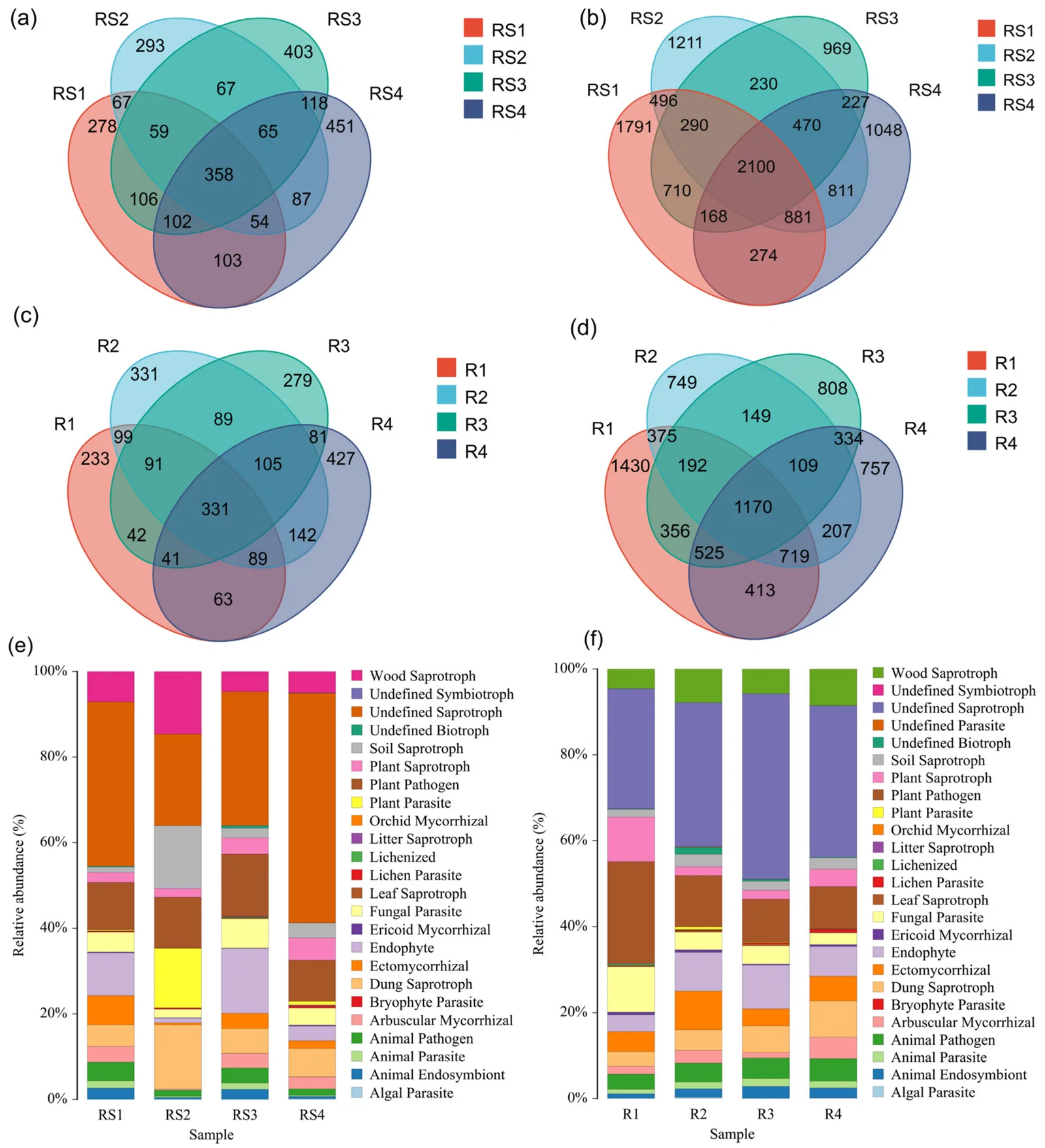
Analysis of microbial communities in roots and rhizosphere soil of *A. mongholicus*. (**a**) Venn diagram of fungal OTUs in rhizosphere soil; (**b**) Venn diagram of bacterial OTUs in rhizosphere soil; (**c**) Venn diagram of fungal OTUs in roots; (**d**) Venn diagram of bacterial OTUs in roots; FunGuild prediction of fungal functional groups in the rhizosphere soil (**e**) and roots (**f**) of *A. mongholicus* across different cultivation years.

**Figure 3:**
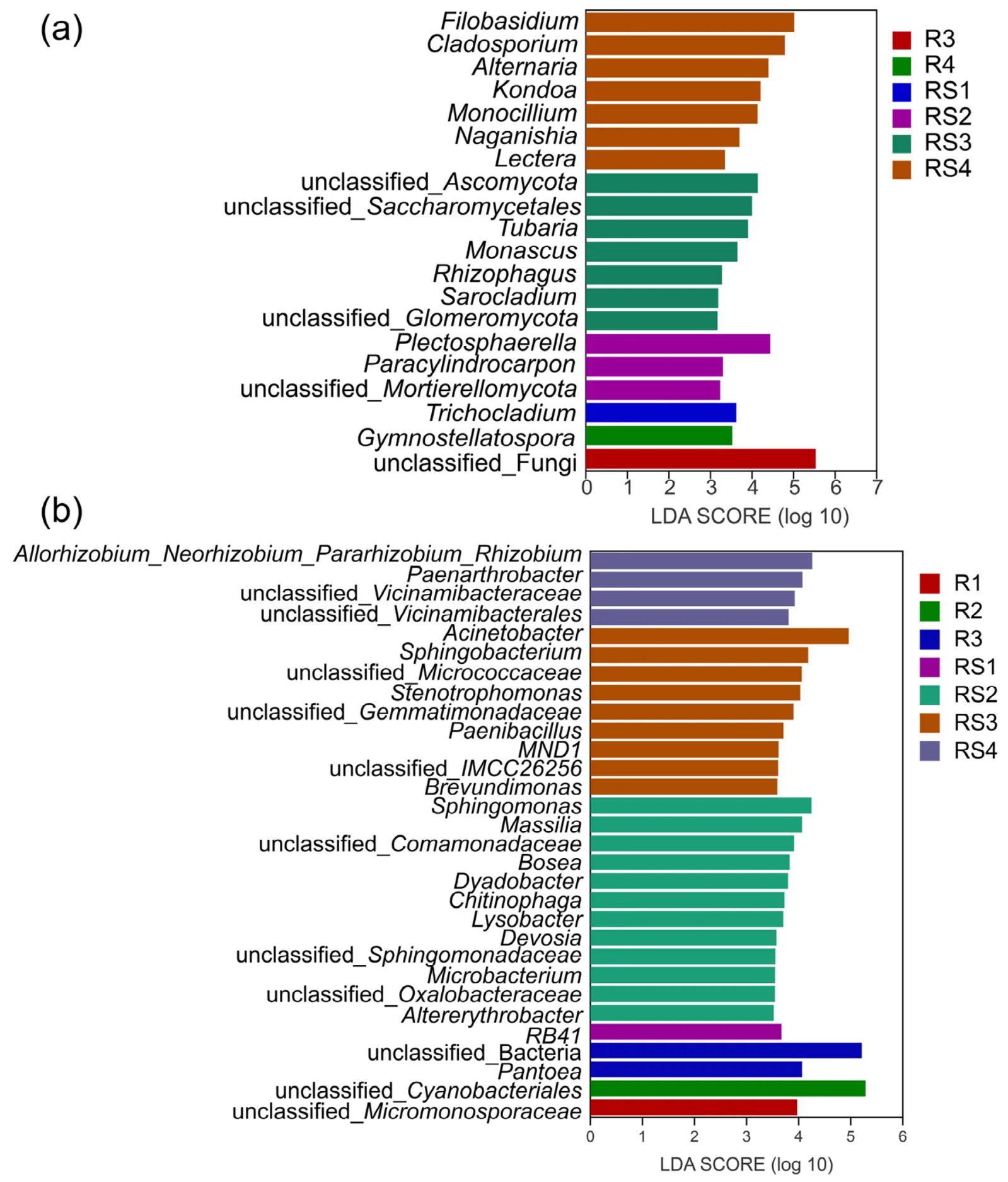
LEfSe analysis of rhizosphere microbial communities of *A. mongholicus* across different cultivation years: (**a**) fungi; (**b**) bacteria.

A Venn diagram of OTUs showed that the unique fungal OTUs in rhizosphere soil increased with longer cultivation duration. For *A. mongholicus* of the same cultivation year, the unique bacterial OTUs in rhizosphere soil were consistently higher than those in roots, and the highest number of unique bacterial OTUs was observed in RS1 and R1 (Figure 2). Based on LEfSe with an LDA score > 3.5, 30 biomarker genera were identified among bacterial communities in rhizosphere soil and roots of *A. mongholicus* across different cultivation years (Figure 3b). Excluding unclassified taxa, *Pantoea* was identified as a biomarker in R3. Rhizosphere biomarkers varied by cultivation stage: RB41 for RS1; *Sphingomonas*, *Massilia*, *Bosea*, *Dyadobacter*, *Chitinophaga*, *Lysobacter*, and others for RS2; *Acinetobacter*, *Sphingobacterium*, *Stenotrophomonas*, and others for RS3; and *Allorhizobium–Neorhizobium–Pararhizobium–Rhizobium* and *Paenarthrobacter* for RS4. For fungi, LEfSe analysis (LDA score > 3.0) identified 20 biomarker genera from roots and rhizosphere (Figure 3a). Among these, *Gymnostellatospora* characterized R4; *Trichocladium* characterized RS1; *Plectosphaerella* and *Paracylindrocarpon* characterized RS2; *Tubaria*, *Monascus*, *Rhizophagus*, and *Sarocladium* characterized RS3; and *Filobasidium*, *Cladosporium*, *Alternaria*, and others characterized RS4. Collectively, the LEfSe analysis reveals marked disparities in the biomarker taxa across the rhizosphere groups under different cultivation years.

FUNGuild predicted distinct ecological functions of rhizosphere fungal communities in *A. mongholicus* across cultivation years (Figure 2e,f). With rising cultivation age, the relative abundance of plant pathogens in root samples (R) progressively decreased from 23.37% to 11.96%, 10.00%, and 9.95%, while dung saprotrophs gradually increased from 3.46% to 4.72%, 6.20%, and 8.38%. Endophytes were more abundant in RS1 and RS3 than in RS2 and RS4, and RS2 had high proportions of soil saprotrophs, plant parasites, and dung saprotrophs. FAPROTAX functional analysis showed that heterotrophic-metabolism-related functions (fermentation, aerobic chemoheterotrophy, chemoheterotrophy) peaked in R1 and exhibited an overall trend of initial decrease followed by an increase as cultivation age advanced (Figure S4). The relative abundance of microbial groups related to phototrophic and photoautotrophic functions (photoautotrophy, oxygenic photoautotrophy, phototrophy, and cyanobacteria) was highest in RS1, while functional groups of aromatic compound degradation were enriched in RS2, RS3 and RS4.

### 2.4. Soil Physicochemical Characteristics and Environmental Drivers of Microbial Community Variation

**Figure 4:**
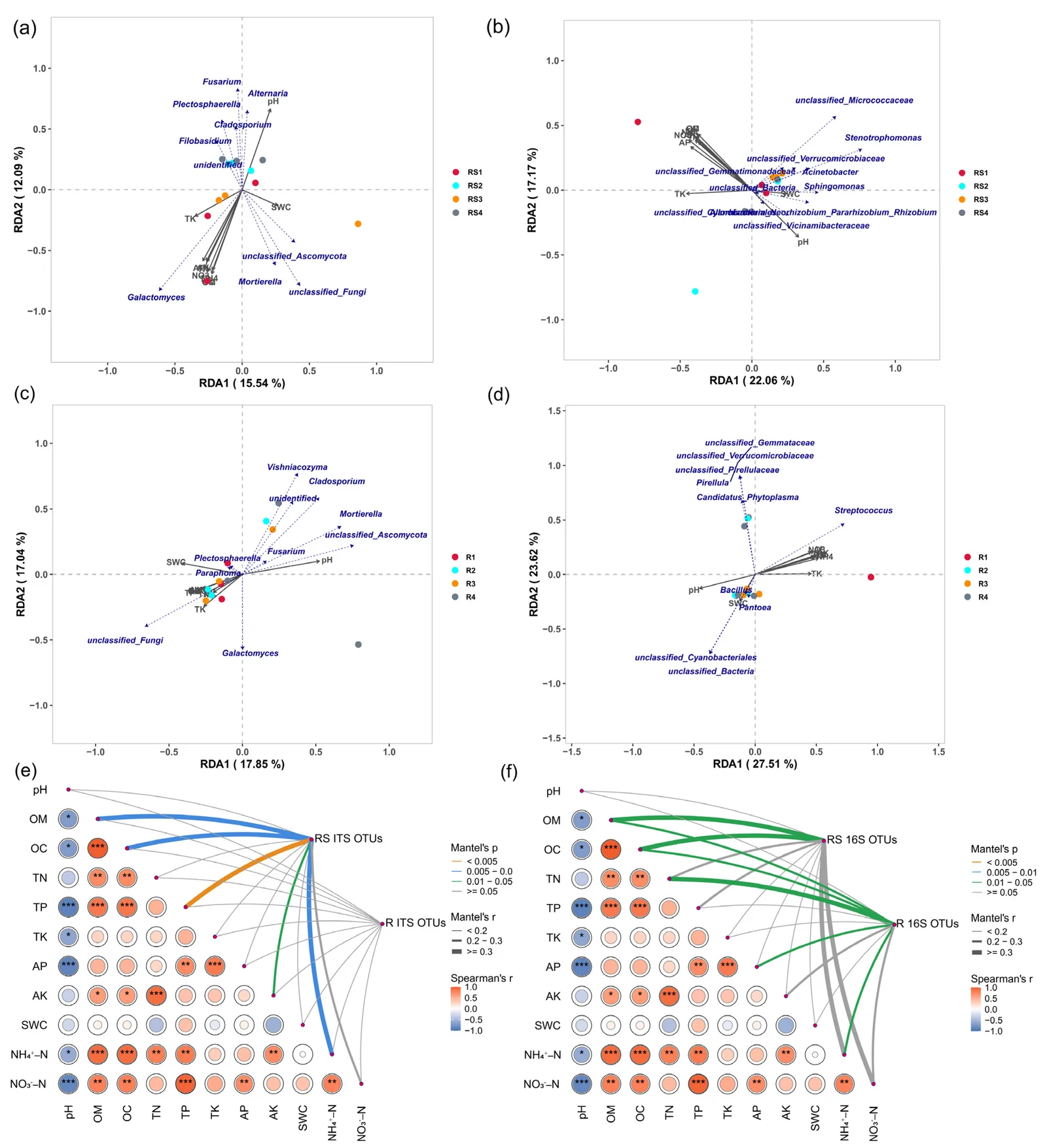
Effects of soil environmental variables on the dominant fungal and bacterial communities in rhizosphere soil and roots of *A. mongholicus*. RDA ordination of the relationships between soil environmental variables and genus composition of (**a**) rhizosphere soil fungi, (**b**) rhizosphere soil bacteria, (**c**) root fungi, and (**d**) root bacteria. Mantel test analysis of the correlations between soil environmental factors and the community composition of (**e**) fungal and (**f**) bacterial communities. *, **, *** indicate *p* < 0.05, 0.01, 0.001, respectively.

The basic physicochemical properties of soils collected from *A. mongholicus* across different cultivation durations are presented in Table 1. With increasing cultivation duration, soil pH generally increased, whereas most soil nutrient parameters declined: OM, TP, TN, TK, OC, AP, AK, NH_4_^+^-N, and NO_3_^−^-N were highest in the 1YR and generally decreased thereafter. SWC was highest in 3YR and lowest in 4YR. We performed redundancy analysis (RDA) to assess the effects of soil physicochemical factors on microbial community structures (Figure 4). In rhizosphere soil, the RDA model explained 27.63% of the cumulative variance for fungal communities and 39.23% for bacterial communities. OC (*r*^2^ = 0.5289, *p* = 0.03), OM (*r*^2^ = 0.5287, *p* = 0.03), and TP (*r*^2^ = 0.5087, *p* = 0.035) were the principal factors determining the distribution of fungal genera in RS samples. In rhizosphere soil, fungal genera *Fusarium*, *Alternaria*, *Cladosporium*, *Plectosphaerella*, and *Filobasidium* were positively correlated with soil pH, while *Galactomyces* and *Mortierella* were positively associated with TK, AP, and OM. In root samples, fungal genera *Vishniacozyma*, *Cladosporium*, *Mortierella*, and *Fusarium* were positively associated with soil pH, whereas *Paraphoma* and *Plectosphaerella* were more strongly associated with environments characterized by high SWC, TK, OM, TN, and AP. Whether in the rhizosphere soil or in the root samples, *Fusarium* and *Cladosporium* were negatively correlated with OC, OM, and TP. For bacteria, unclassified Vicinamibacteraceae, unclassified Micrococcaceae, *Stenotrophomonas*, *Sphingomonas*, *Acinetobacter*, and *Allorhizobium–Neorhizobium–Pararhizobium–Rhizobium* were positively associated with pH and negatively correlated with soil nutrients such as OC, OM, and TP. In root samples, *Streptococcus* was positively correlated with soil nutrient variables, while *Bacillus*, *Pantoea*, unclassified Cyanobacteriales and unclassified Bacteria were positively associated with pH and SWC. The Mantel test indicated that OM, OC, and nutrient factors, including NH_4_^+^-N, N, P, and K, may represent important environmental variables associated with shifts in the microbial community composition of *A. mongholicus* (Figure 4e,f).

### 2.5. Variations in Bioactive Compound Contents and Their Correlations with Microbial Communities in A. mongholicus at Different Cultivation Durations

**Figure 5:**
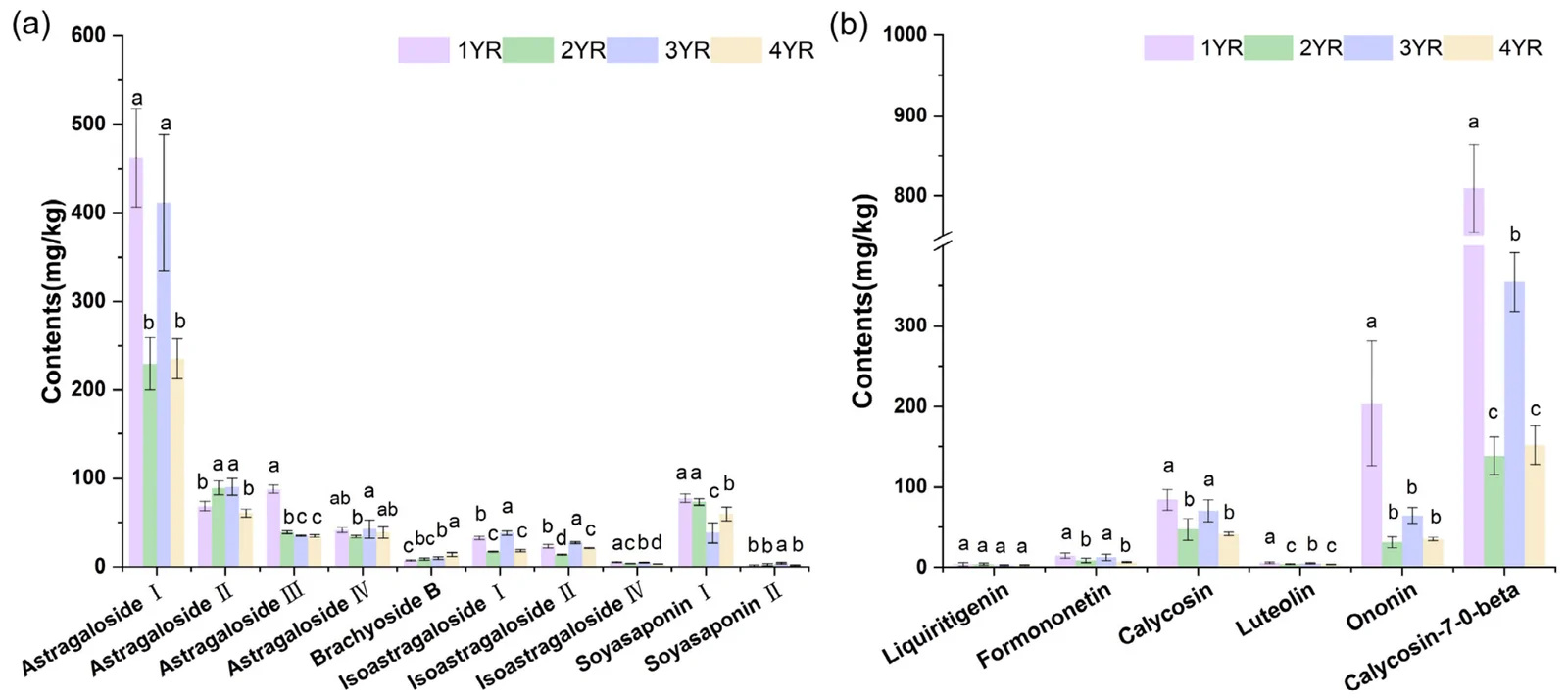
Determination of the content of active substances in *A. mongholicus* roots: (**a**) triterpenoid saponins; (**b**) flavonoids. 1YR, 2YR, 3YR, and 4YR represent root samples from 1-, 2-, 3-, and 4-year-old *A. mongholicus*, respectively. Different lowercase letters (a–d) indicate significant differences among groups (*p* < 0.05).

**Figure 6:**
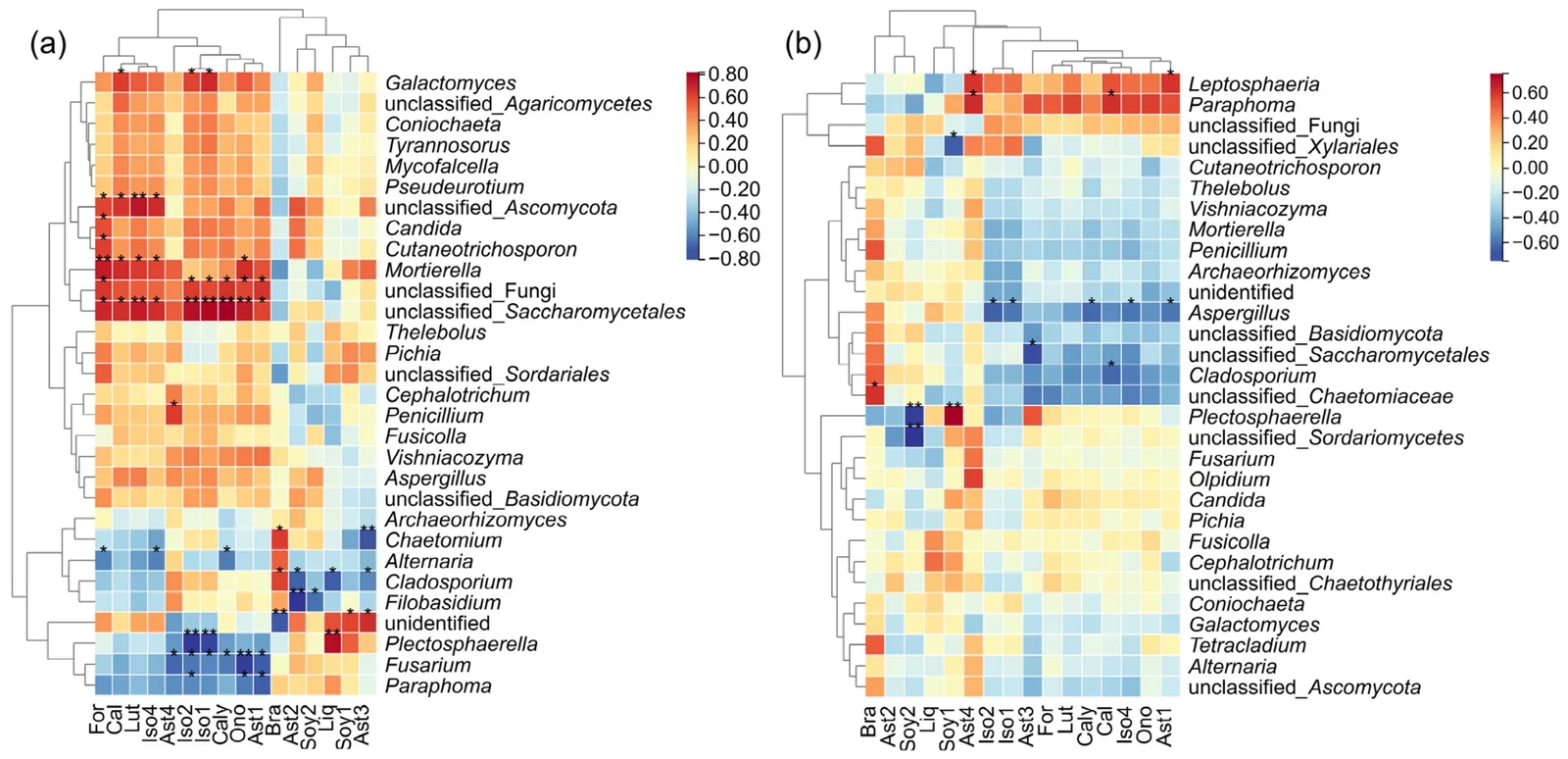
Heatmap of the correlation between active components of *A. mongholicus* and fungal microbial communities ((**a**) rhizosphere soil; (**b**) roots). Orange indicates a positive correlation, blue indicates a negative correlation, and the darker the color, the higher the correlation (|r| > 0.6); *, and ** indicate *p* < 0.05, and 0.01, respectively. Ast2: Astragaloside II, Bra: Brachyoside B, Ast4: Astragaloside IV, Liq: Liquiritigenin, Cal: Calycosin, Ono: Ononin, Caly: Calycosin-7-O-beta-D-glucoside, Ast1: Astragaloside I, Soy2: Soyasaponin II, Ast3: AstragalosideIII, Soy1: Soyasaponin I, Iso1: Isoastragaloside I, Iso2: Isoastragaloside II, Iso4: Isoastragaloside IV, Lue: Luteolin, For: Formononetin.

**Figure 7:**
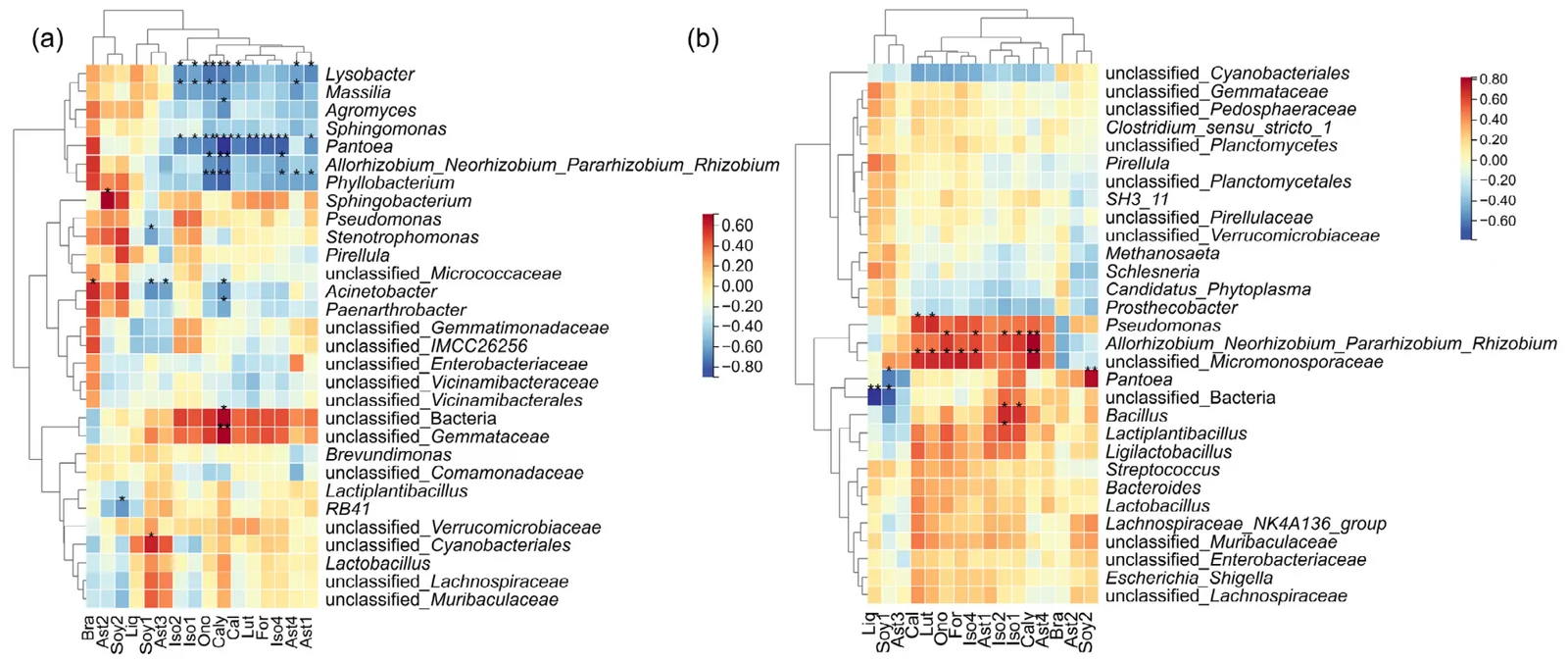
Heatmap of the correlation between active components of *A. mongholicus* and bacterial microbial communities ((**a**) rhizosphere soil; (**b**) roots). Orange indicates a positive correlation, blue indicates a negative correlation, and the darker the color, the higher the correlation (|r| > 0.6); *, **, and *** indicate *p* < 0.05, 0.01, and 0.001, respectively. Ast2: Astragaloside II, Bra: Brachyoside B, Ast4: Astragaloside IV, Liq: Liquiritigenin, Cal: Calycosin, Ono: Ononin, Caly: Calycosin-7-O-beta-D-glucoside, Ast1: Astragaloside I, Soy2: Soyasaponin II, Ast3: Astragaloside III, Soy1: Soyasaponin I, Iso1: Isoastragaloside I, Iso2: Isoastragaloside II, Iso4: Isoastragaloside IV, Lue: Luteolin, For: Formononetin.

The contents of 16 bioactive compounds in the roots of 1- to 4-year-old *A. mongholicus* plants were quantified (Table S4). Significant differences in saponin and flavonoid contents were observed among cultivation years (Figure 5). Ononin, calycosin-7-O-β-D-glucoside, astragaloside III, and luteolin were highest in first-year plants, whereas astragaloside II, astragaloside IV, soyasaponin II, isoastragaloside I, and isoastragaloside II peaked in third-year plants. Calycosin and astragaloside I showed higher contents in first- and third-year plants than in second- and fourth-year plants. Brachyoside B increased progressively from 7.29 to 13.87 mg kg^−1^, whereas astragaloside III decreased from 87.80 to 34.76 mg kg^−1^ with increasing cultivation duration. Liquiritigenin did not differ significantly among cultivation years. Correlations between active-constituent contents and the 30 most abundant microbial genera were analyzed in rhizosphere soil and roots (Figure 6 and Figure 7). In rhizosphere soil, *Galactomyces* and *Mortierella* were positively correlated with several flavonoids and saponins, whereas *Fusarium*, *Paraphoma*, and *Plectosphaerella* showed predominantly negative correlations. In roots, *Leptosphaeria* and *Paraphoma* were positively associated with several compounds, while *Aspergillus* showed predominantly negative correlations. Bacterial associations were also compartment-specific. In rhizosphere soil, *Lysobacter*, *Massilia*, *Pantoea*, *Phyllobacterium*, and the *Allorhizobium–Neorhizobium–Pararhizobium–Rhizobium* group were negatively correlated with several flavonoids and saponins, whereas *Sphingobacterium* showed a positive association with astragaloside II. In roots, *Pseudomonas*, *Bacillus*, and the *Allorhizobium–Neorhizobium–Pararhizobium–Rhizobium* group were positively associated with several active constituents, while *Pantoea* showed opposite correlations with soyasaponin I and II. Overall, microbial communities in rhizosphere soil and *A. mongholicus* roots cultivated for different durations showed significant correlations with multiple flavonoid and saponin constituents. Certain microbial taxa, including *Mortierella*, *Pseudomonas*, *Bacillus*, and the *Allorhizobium–Neorhizobium–Pararhizobium–Rhizobium* group, may be closely associated with the accumulation of bioactive constituents in *A*. *mongholicus*. In contrast, genera such as *Fusarium* and *Aspergillus* were negatively associated with the accumulation of several active constituents.

## 3. Discussion

### 3.1. Effects of Cultivation Year on Rhizosphere Microbial Community

Previous studies have demonstrated complex, interdependent, regulatory, and mutually constraining interactions among plants, rhizosphere microorganisms, and the environment [2]. In the present study, rhizosphere fungal community diversity differed among *A. mongholicus* plants cultivated for different durations. In contrast, the Chao1 and Shannon indices of bacterial communities showed only minor variations among different cultivation years, suggesting that bacterial diversity was less sensitive to cultivation duration than fungal diversity under the current sampling design. This may reflect differences in soil pH, as soil pH and physicochemical properties are closely associated with bacterial microbial diversity [29,30,31]. Continuous cropping in neutral soil did not significantly affect bacterial community diversity [29]. However, continuous cropping under acidic conditions may change soil microbial diversity [32]. The soil pH ranged from 8.17 to 8.53 in this study, indicating weakly alkaline conditions. Although the Shannon index exhibited no statistically significant inter-group variation, planting duration altered the relative abundances of bacterial communities, with such effects mainly reflected in shifts in community composition and dominant taxa. Furthermore, given the limited sample size (*n* = 3 per year), this should not be interpreted as evidence of stability and is a pattern that warrants verification in future studies with larger cohorts.

In this study, the unique fungal OTUs in rhizosphere soil increased with increasing cultivation duration, and, within each cultivation year, rhizosphere soil consistently contained more unique bacterial OTUs than the roots. These findings suggest that the rhizosphere microbiome does not become progressively homogenized from the seedling stage to mature plants; instead, it undergoes continuous environmental filtering over time, enriching an increasing number of stage-specific fungal taxa [33]. These exclusive fungal taxa may form distinct functional guilds mediating nutrient cycling and plant–fungal interactions [34]. Nevertheless, this accumulation may also raise the risk of enrichment of harmful fungal taxa [35]. Meanwhile, the rhizosphere may serve as a reservoir of microbial diversity, consistently harboring a larger pool of unique taxa than the roots [36]. The declining predicted relative abundance of plant pathogens, coupled with higher dung saprotroph proportions in roots, indicates that extended cultivation periods might restructure the functional profiles of root fungal assemblages, potentially shifting the community from plant pathogen-associated guilds toward saprotrophic guilds with putative organic-matter-decomposition capabilities. Taxa classified as plant pathogen-associated were interpreted as microorganisms previously reported to be associated with plant diseases and potentially relevant to root–microbe interactions [37]. Their presence in rhizosphere or root-associated samples does not necessarily indicate active infection or pathogenicity in *A. mongholicus*. With increasing cultivation duration, accumulated root exudates may provide more substrates for saprotrophic guilds and niche occupation. Nutrient competition and potential antagonistic effects of saprophytic guilds may further inhibit the colonization of pathogens [38]. Furthermore, accumulating inhibitory microorganisms may also reduce pathogen abundance [39,40]. Notably, the relative abundances of Mortierellomycota and *Mortierella* gradually declined in rhizosphere soil with increasing cultivation duration. Meanwhile, potentially phytopathogenic genera, including *Fusarium*, *Alternaria*, and *Cladosporium*, showed relatively high relative abundances in RS4. *Mortierella* is considered a potentially beneficial fungal genus in agricultural soils and has been reported to promote plant growth [41]. In line with our present observations, Zhang et al. [42] found that the relative abundance of *Mortierella* gradually decreased with prolonged cultivation. In addition, the relative abundances of *Allorhizobium–Neorhizobium–Pararhizobium–Rhizobium* and *Pseudomonas* were relatively high in R1. The enrichment of rhizosphere bacteria with potential nitrogen-fixing, phytohormone-producing, and phosphate-solubilizing capacities may facilitate the early assembly of the root-associated microbiome and support the establishment and environmental adaptation of young plants [43,44,45]. *Pantoea* was relatively abundant in R3 and served as a specific indicator genus in this sample group. Previous studies have shown that the endophytic strain *Pantoea* sp. YSD J2 exhibits a range of functions facilitating plant growth and metabolic processes [46]. The microbial communities in the roots were analyzed in our study, although external contaminants could not be excluded. A small portion of DNA from microbial communities present on the tissue surface may remain even after surface sterilization and several washes [47]. Although this caveat is unlikely to alter the major community patterns reported herein, it should be kept in mind when interpreting differences between root endosphere and rhizosphere microbiomes.

### 3.2. Soil Physicochemical Properties Driving Microbial Community Variation

Soil physicochemical properties reflect soil nutrient and health status and are dominant factors affecting the diversity and dynamics of rhizosphere microbial communities [48,49]. In this study, with increasing cultivation duration, soil pH gradually increased (*p* < 0.05), whereas the basic soil physicochemical properties were highest in the first year, consistent with Li et al. [16]. AP, AK, and NO_3_^−^-N were significantly higher in the first-year soil but gradually decreased thereafter. This pattern may be ascribed to the application of organic fertilizer before *A. mongholicus* transplantation, as well as to the progressive decline in the soil microbial community’s capacity to utilize nitrogen, sulfur, and potassium [50,51]. In the rhizosphere fungal community, genera such as *Fusarium*, *Alternaria*, *Cladosporium*, *Plectosphaerella*, and *Filobasidium* were positively associated with soil pH, indicating that soil pH may act as an important environmental filter shaping the rhizosphere fungal community [52]. In contrast, *Galactomyces*, *Mortierella*, and other fungal taxa were positively associated with soil physicochemical characteristics, including TK, AP, and OM, suggesting that organic matter and nutrient availability may provide more advantageous circumstances for the colonization, growth, and potential ecological functions of potentially beneficial fungi such as *Mortierella* [53,54]. In the root samples, *Vishniacozyma*, *Cladosporium*, and *Fusarium* were likewise positively associated with soil pH. However, some differences were observed: *Mortierella* was positively associated with pH, whereas *Paraphoma* and *Plectosphaerella* were preferentially associated with conditions characterized by higher SWC, TK, OM, TN, and AP. These findings suggest that root-associated microorganisms are not merely passive reflections of the surrounding soil environment; root exudates, plant immune responses, and plant developmental processes may also shape their distribution [55,56,57].

### 3.3. Variations in Bioactive Compounds with Cultivation Years and Their Relationships with Microbial Communities

Previous studies show that the contents of major bioactive compounds in *A. mongholicus*, including astragaloside IV and calycosin-7-glucoside, vary dynamically with cultivation duration, thereby affecting the quality and chemical composition of the medicinal material [24,58]. Although the specific patterns of variation remain inconsistent among studies, most reports suggest that plants cultivated for two to three years accumulate relatively high levels of astragaloside IV [24,59]. In the current study, the amounts of saponins and flavonoids in *A. mongholicus* roots varied significantly among plants of different cultivation ages. The contents of calycosin, calycosin-7-O-glucoside, astragaloside I, and isoastragaloside I exhibited a decrease–increase–decrease pattern across the cultivation years. Ononin, calycosin-7-O-glucoside, astragaloside III, and luteolin showed the highest contents in one-year-old plants, whereas astragaloside II, astragaloside IV, soyasaponin II, isoastragaloside I, and isoastragaloside II reached their maximum levels in three-year-old plants. A similar pattern has been observed in *Bupleurum scorzonerifolium*, where total saponin content was highest in younger plants (1 growth year) and decreased significantly from 1 to 2 growth years, then increased at 3 growth years [60]. This may be due to the plant’s early growth stage, which is characterized by thinner cork layers and lower levels of structural and defense-related cell-wall components, and prioritizes synthesizing defensive bioactive compounds such as saponins to improve environmental stress adaptability [60]. Beyond this early stress response, a metabolic-accumulation effect likely accounts for the recovery observed in year 3. Many specialized metabolites (e.g., saponins and certain flavonoids) are continuously synthesized and gradually deposited in root tissues [61]. The combination of sustained biosynthesis and relatively low degradation or turnover thus drives the rebound of these compounds, particularly between the second and third growing seasons.

*Mortierella*, *Pseudomonas*, *Bacillus*, and *Allorhizobium–Neorhizobium–Pararhizobium–Rhizobium* may be closely associated with the buildup of bioactive compounds such as calycosin and ononin in *A. mongholicus*, whereas *Fusarium* and *Aspergillus* showed negative correlations with astragaloside I, isoastragaloside I, and isoastragaloside II. *Mortierella* is considered a beneficial microorganism because of its ability to solubilize phosphorus and decompose organic matter, and it has been proposed as a potential biofertilizer to promote plant growth [62]. Plant growth-promoting rhizobacteria, including *Bacillus* spp., *Pseudomonas* spp., and *Rhizobium* spp., can colonize the rhizosphere and facilitate plant growth and nutrient uptake through microbial nitrogen fixation, soil nutrient solubilization, and the synthesis of plant growth regulators, like indole-3-acetic acid (IAA), cytokinins, and gibberellins (GAs) [63]. These findings suggest that rhizosphere and root-associated microorganisms may contribute to the increased levels of bioactive compounds and the formation of *A. mongholicus* quality. Nevertheless, these results do not establish a direct biosynthetic relationship. The genera identified here should be regarded as candidate ecological correlates, and their ability to produce or transform the corresponding compounds requires direct experimental validation.

## 4. Materials and Methods

### 4.1. Sampling

In June 2023, *A. mongholicus* plants aged 1–4 years were collected from a mountain-transplanted cultivation field in Sishilipu Town, Suide County, Yulin City, Shaanxi Province, China (37°40′21″ N, 110°10′48″ E), managed by Suide Qiangsheng Agricultural Technology Co., Ltd. (Yulin, China). For each cultivation age, three 2 m × 2 m plots were randomly selected, and three healthy plants were sampled per plot. One-year-old plants were sown in 2022 and transplanted in spring 2023; two-, three-, and four-year-old plants were sown in 2021, 2020, and 2019, respectively, and transplanted in spring 2022, 2021, and 2020, respectively. For rhizosphere soil collection, each plant was excavated with a shovel and gently shaken to remove loosely attached soil. Soil adhering tightly to the roots was brushed into sterile bags and defined as rhizosphere soil according to Huo et al. [64]; approximately 50 g was collected per plant. Non-rhizosphere soil was collected after plant removal from a depth of approximately 20 cm and a distance of 5 cm from the main root, with approximately 300 g collected per plant. Roots were placed in sterile bags and labeled. All samples were transported to the laboratory within 3 days. Soil was sieved to remove stones, roots, and debris. Roots were rinsed, surface-disinfected with 75% ethanol for 5 min, rinsed three times with sterile water, treated with 2% NaClO for 5 min, and thoroughly rinsed again. Processed soil and root samples were transferred to sterile 5-mL centrifuge tubes, rapidly frozen in liquid nitrogen, and stored at −80 °C. Approximately 1.5 kg of surface soil was also collected from each planting area at a depth of 0–20 cm for physicochemical analysis.

### 4.2. Measurement of Soil Physicochemical Properties

The collected soil samples were sieved to remove plant debris and gravel particles, then sent to Pony Testing International Group (Beijing, China) for analysis. The parameters tested included: pH, organic matter (OM), total phosphorus (TP), total nitrogen (TN), total potassium (TK), organic carbon (OC), available phosphorus (AP), soil water content (SWC), available potassium (AK), nitrate nitrogen (NO_3_^−^-N) and ammonium nitrogen (NH_4_^+^-N). The specific analytical methods are listed in Table S1.

### 4.3. DNA Extraction, Amplicon Amplification and Illumina Sequencing

Total genomic DNA was extracted using the TGuideS96 magnetic bead-based soil/stool genomic DNA extraction kit (Tiangen Biotech, Beijing, China; Model: DP812) following the manufacturer’s instructions. DNA content and purity were determined using a microplate reader (Gene Company Limited, Model: Synergy HTX, Chai Wan, Hong Kong). Amplification was subsequently carried out, and the resulting products were subjected to electrophoresis on a 1.8% agarose gel (Beijing Bomeifuxin Technology Co., Ltd. Beijing, China) to assess integrity. Briefly, the V3–V4 region of the bacterial 16S rRNA gene was amplified using primers 338F (5′-ACTCCTACGGGAGGCAGCA-3′) and 806R (5′-GGACTACHVGGGTWTCTAAT-3′) [65]. The internal transcribed spacer (ITS) region of fungi was amplified with primers ITS1F (5′-CTTGGTCATTTAGAGGAAGTAA-3′) and ITS2R (5′-GCTGCGTTCTTCATCGATGC-3′) [66]. After PCR amplification, purification, and sample quantification, the library was subsequently sequenced on the Illumina NovaSeq 6000 platform (Beijing Biomarker Technologies Co., Ltd., Beijing, China). Sequences were clustered with USEARCH v10.0 using a 97% sequence-similarity cutoff [67]. OTUs (Operational Taxonomic Units) accounting for less than 0.005% of total sequencing reads were removed as a filtering step [68].

### 4.4. Reagents and Plant Materials

The reference chemicals astragaloside I (99.2%), astragaloside II (99.9%), astragaloside III (99.7%), astragaloside IV (99.5%), daidzein (97.0%), formononetin (98.7%), brachyoside B (99.3%), isoastragaloside IV (99.9%), isoastragaloside I (99.7%), isoastragaloside II (99.1%), soyasaponin II (99.4%), soyasaponin I (99.4%), kaempferol 3-O-robinobioside (99.9%), apigenin-7-O-β-D-glucoside (98.4%), liquiritigenin (97.7%), luteolin (99.2%), orientin (98.8%), quercitrin (98.0%), salvigenin (99.9%), vitexin (99.9%), and apigenin-7-O-(2G-rhamnosyl) gentiobioside (99.9%) were purchased from Tianjin Alta Technology (Tianjin, China). The reference chemicals 3-hydroxyflavone, 7-hydroxyflavone, apigenin, astragalin, calycosin, calycosin-7-O-β-D-glucoside, hyperoside, isorhamnetin, mangiferin, narcissoside, naringin, ononin, rutin and wogonin were solid reagents with purity greater than 98% (Table S2). The root samples of *A. mongholicus* plants aged 1–4 years assayed for bioactive compounds were sourced from the collections in Section 2.1. After collection, the roots were dried at 50 °C.

### 4.5. Determination of Bioactive Compound Contents

An Xevo TQ-XS ultra-performance liquid chromatography–triple quadrupole tandem mass spectrometry system (Waters, Milford, MA, USA) was used for compound detection and analysis. Chromatographic separation was carried out using a CORTECS^®^ UPLC^®^ C18 column (1.6 μm, 2.1 mm × 100 mm; Waters, USA). Sample preparation and instrument parameters are provided in the Supplementary Information. We qualitatively identified compounds by comparing retention times and ion-pair information with those of the corresponding standards. Quantification was conducted using the precursor ions and the product ions exhibiting the highest response. Table 2 presents the specific mass spectrometric parameters for each compound. The mixed calibration standard solution was prepared by combining saponin and flavonoid standard samples. This mixed standard stock solution was serially diluted with methanol to produce working standard solutions across a series of appropriate concentrations within the ranges [27,28]. By comparing the retention times and peak areas with the standard calibration curve (Table S3), the target compounds were identified and quantified using the external standard method.

### 4.6. Data Statistics and Analysis

The α-diversity indices, including the Shannon and Chao1 indices, were calculated for bacterial and fungal communities using a t-test for inter-group variance analysis. Non-metric multidimensional scaling (NMDS) based on β–Curtis dissimilarities was performed to assess differences in bacterial and fungal community composition. Using Bray–Curtis distance, PERMANOVA was performed to assess differences between sample groups with different planting years. FAPROTAX v1.2.6 databases were used to predict the ecological function information of bacterial communities [69]. The FUNGuild v1.0 database was used to analyze the ecological function information of fungal communities [37]. Biomarkers were identified through linear discriminant analysis (LDA; LDA score > 3.5 for bacteria and LDA score > 3.0 for fungi). Mantel test was used to explore the correlations between microbial communities and soil physicochemical properties using the “LinkET” package in R.

## 5. Conclusions

Under mountain-transplanted cultivation, increasing cultivation duration in *A. mongholicus* was accompanied by rising soil pH and declining organic matter and nutrient availability. These soil shifts were associated with microbial community succession, particularly changes in fungal diversity and composition, including declining relative abundances of Mortierellomycota and *Mortierella* and increasing numbers of year-specific fungal OTUs. Bacterial α-diversity did not differ significantly among cultivation years, although bacterial taxonomic composition varied. The positive associations of rhizosphere *Mortierella* and *Galactomyces* with organic matter and nutrient availability, together with their correlations with several root flavonoids and saponins, suggest a potential connection between soil conditions, fungal community composition, and bioactive compound accumulation. Root-associated Pseudomonas, Bacillus, and the *Allorhizobium–Neorhizobium–Pararhizobium–Rhizobium* complex also showed positive associations with several bioactive constituents. Compound accumulation followed distinct cultivation-year-dependent patterns: several flavonoids and astragaloside III peaked in first-year plants, whereas astragalosides II and IV and several other saponins peaked in third-year plants. Collectively, these findings indicate that variation in medicinal constituent contents across cultivation years should be interpreted alongside concurrent changes in soil fertility and the rhizosphere and root-associated microbiomes. Although these associations do not establish microbial biosynthesis or causal effects on plant metabolism, they identify soil fertility maintenance and selected microbial taxa as priorities for future experiments aimed at improving cultivation management and medicinal quality.

## Figures and Tables

**Table 1:** Soil physicochemical properties under different cultivation years.

Physicochemical Properties	1YR	2YR	3YR	4YR
SWC (%)	7.30 ± 0.1 ^c^	9.60 ± 0.1 ^b^	10.33 ± 0.12 ^a^	6.00 ± 0.1 ^d^
pH	8.17 ± 0.01 ^c^	8.38 ± 0.01 ^b^	8.38 ± 0.01 ^b^	8.53 ± 0.01 ^a^
OM (g/kg)	7.29 ± 0.06 ^a^	2.73 ± 0.10 ^c^	3.58 ± 0.07 ^b^	2.78 ± 0.07 ^c^
TP (g/kg)	0.84 ± 0.006 ^a^	0.61 ± 0.01 ^c^	0.63 ± 0.01 ^b^	0.59 ± 0.01 ^d^
TN (g/kg)	0.64 ± 0.02 ^a^	0.13 ± 0.01 ^c^	0.16 ± 0.01 ^b^	0.16 ± 0.01 ^b^
TK (g/kg)	19.03 ± 0.15 ^a^	18.73 ± 0.15 ^ab^	18.23 ± 0.15 ^c^	18.37 ± 0.32 ^bc^
OC (g/kg)	4.23 ± 0.04 ^a^	1.58 ± 0.06 ^c^	2.07 ± 0.04 ^b^	1.62 ± 0.04 ^c^
AP (mg/kg)	20.03 ± 0.15 ^a^	2.00 ± 0.00 ^b^	0.37 ± 0.06 ^c^	0.30 ± 0.00 ^c^
AK (mg/kg)	113.67 ± 0.08 ^a^	51.00 ± 0.00 ^d^	57.33 ± 0.58 ^c^	62.00 ± 1.00 ^b^
NH_4_^+^-N (mg/kg)	1.44 ± 0.04 ^a^	0.67 ± 0.02 ^c^	0.79 ± 0.03 ^b^	0.70 ± 0.01 ^c^
NO_3_^−^-N (mg/kg)	13.07 ± 0.47 ^a^	1.29 ± 0.04 ^b^	1.58 ± 0.06 ^b^	1.17 ± 0.04 ^b^

^a,b,c,d^ different superscripts in the same row represent significant differences (*p* < 0.05).

**Table 2:** MS/MS parameters of the active substances under the MRM ESI mode.

Compound Name	RT (min)	Precursor Ion (*m*/*z*)	Product Ions (*m*/*z*)	Cone Energy (V)	Collision Energy (V)
3-Hydroxyflavone	6.23	239.14	121.07 *	46	26
			165.06	46	28
7-Hydroxyflavone	5.57	239.14	137.02 *	56	26
			129.08	56	26
Apigenin	5.35	271.08	153.06 *	20	26
			119.1	20	28
Apigenin-7-O-(2G-rhaMnosyl) gentiobioside	4.18	741.25	271.16 *	22	34
			579.21	22	16
Apigenin-7-O-β-D-glucoside	4.56	433.16	271.11 *	24	16
			119.1	24	50
Astragalin	4.63	449.16	85.06	12	22
			287.11 *	12	10
Astragaloside II	6.39	849.4	203.12 *	100	56
			477.37	100	52
Astragaloside III	6.34	786.32	142.95 *	10	22
			106.74	15	20
Astragaloside IV	6.36	807.43	627.37 *	100	50
			203.11	100	54
Astragaloside I	6.56	869.63	143.16 *	6	14
			217.1	15	15
Brachyoside B	6.45	675.38	203.11 *	100	48
			107.16	100	60
Calycosin	4.99	284.993	225 *	2	20
			212.9	2	20
Calycosin-7-O-beta-D-glucoside	4.14	447.019	285 *	2	30
			269.9	2	30
Daidzein	4.88	255.08	199.16 *	64	22
			137.08	64	24
Formononetin	5.51	269.1	197.12 *	2	36
			237.13	2	26
Hyperoside	4.40	465.09	303.11 *	30	12
			153.07	30	48
Isoastragaloside IV	6.01	785.438	437.419	12	18
			143.16 *	12	16
Isoastragaloside I	6.65	869.699	139.128 *	18	30
			143.162	18	12
Isoastragaloside II	6.53	828.255	143.2 *	10	25
			438.3	10	25
Isorhamnetin	5.35	317.1	153.06	44	30
			302.12 *	44	26
Kaempferol 3-O-robinobioside	4.57	595.23	287.08 *	32	18
			449.18	32	8
Liquiritigenin	4.75	257.15	137.08 *	34	22
			147.1	34	18
Luteolin	5.12	287.21	135.09 *	32	30
			153.05	32	30
Mangiferin	3.90	423.12	273.11 *	38	22
			327.14	38	14
Narcissoside	4.67	625.19	317.17 *	32	18
			479.19	32	10
Naringin	4.34	581.25	273.16 *	18	18
			153.05	18	48
Ononin	4.66	431.18	269.14 *	36	12
			253.34	36	46
Orientin	4.07	449.16	329.11 *	22	26
			299.2	22	30
Quercitrin	4.60	449.16	303.11 *	18	10
			85.06	18	18
Rutin	4.37	611.23	303.08 *	32	20
			465.11	32	10
Salvigenin	6.11	329.22	296.1 *	66	22
			268.07	66	28
Soyasaponin II	6.33	913.376	441.391 *	24	22
			423.386	24	32
Soyasaponin I	6.23	943.51	441.44 *	42	24
			423.44	42	28
Vitexin	4.20	433.16	313.12 *	18	24
			283.2	18	30
Wogonin	5.81	285.1	179.12 *	54	34
			270.11	54	26

RT: retention time; *: quantifier ion.

## Data Availability

All raw sequencing data were submitted to the NCBI Sequence Read Archive (SRA) under accession number SRP730226 and linked to NCBI BioProject PRJNA1516729 (https:/www.ncbi.nlm.nih.gov/bioproject/?term=PRJNA1516729).

## Supplementary Materials

The following supporting information can be downloaded at: https://www.mdpi.com/article/10.3390/plants15213493/s1, Figure S1: Analysis of α-diversity (Chao 1 and Shannon index) of fungal communities in non-rhizosphere soil (NRS), rhizosphere soil (RS), and root (R) of *A. mongholicus* from different cultivation years. (a) Bulk soil Chao 1 index; (b) Rhizosphere soil Chao 1 index; (c) Root Chao 1 index; (d) Bulk soil Shannon index; (e) Rhizosphere soil Shannon index; (f) Root Shannon index; Figure S2: Analysis of α-diversity (Chao 1 and Shannon index) of bacterial communities in non-rhizosphere soil (NRS), rhizosphere soil (RS), and root (R) of *A. mongholicus* from different cultivation years. (a) Bulk soil Chao 1 index; (b) Rhizosphere soil Chao 1 index; (c) Root Chao 1 index; (d) Bulk soil Shannon index; (e) Rhizosphere soil Shannon index; (f) Root Shannon index; Figure S3: Phylum-level species composition and relative abundance of fungal (a) and bacterial (b) communities; Figure S4: FAPROTAX functional annotation based on bacterial communities in the rhizosphere soil and root of *A. mongholicus* from different cultivation years; Figure S5: Total ion diagram of 35 active ingredients in standard solution; Figure S6: Quantifier ion chromatograms of 35 bioactive compounds; Table S1: Methods for determining soil physicochemical properties; Table S2: Information on the analytical standards of bioactive compounds; Table S3: Calibration equations for the quantitative determination of flavonoid and saponin constituents in pure solvents; Table S4: The contents of 16 active compounds in the roots of 1- to 4-year-old *A. mongholicus* (mg/kg).
