# Supplementary material for: Dynamics of Rhizosphere Microbial Communities and Bioactive Compound Accumulation in Wild-Simulated Astragalus mongholicus Across Different Cultivation Years
Source: Plants (Basel). 2026 Nov 9;15(21):3493. doi: 10.3390/plants15213493 (PMC13766178; doi:10.3390/plants15213493)
Supplement: Supplementary file 1 [file plants-15-03493-s001.zip › plants-4645488-supplementary.pdf]

Dynamics of Rhizosphere Microbial Communities and Bioactive Compound Accumulation in Wild-Simulated *Astragalus mongholicus* Across Different Cultivation Years

Ouli Xiao [1], Bin Ma [2], Xiaofeng Dai [1], Jieyin Chen [1,3,*], Zhiqiang Kong [1,*]

[1] State Key Laboratory for Biology of Plant Diseases and Insect Pests, Institute of Plant Protection, Chinese Academy of Agricultural Sciences, Beijing 100193, P. R. China

[2] Institute of Forestry and Grassland Ecology, Ningxia Academy of Agricultural and Forestry Sciences, Yinchuan 750002, China

[3] Western Agricultural Research Center, Chinese Academy of Agricultural Sciences, Changji 831100, China

[*]Corresponding author.

E-mail address: chenjieyin@caas.cn

[*]Corresponding author.

E-mail address: kongzhiqiang@caas.cn

[Supplementary material] including 2 paragraphs, 6 figures, and 4 tables.

Materials and methods

1.1 Sample treatment

The extraction procedure for bioactive compounds from *A. mongholicus* roots followed Li et al.[16], with appropriate modifications. Root samples were dried in an oven at 50 °C to constant weight, ground with a high-speed mill, and sieved through a 50-mesh screen. Afterwards, 1.0 g of the obtained powder was placed into a 50 mL centrifuge tube, followed by the addition of 10 mL chromatographic-grade methanol. Ultrasonic extraction was performed for 60 min under 100 W power at 22 °C. Ice was added during sonication when necessary to prevent excessive temperature increases. Following this step, the extract underwent centrifugation at 5000 × g for 10 min to obtain the supernatant. The 1 mL supernatant subsample was filtered with a 0.22 μm membrane filter before instrumental detection.

1.2 LC-MS/MS analytical conditions

The analytes were separated using an ultra-performance LC system (Xevo TQ-XS, Waters, USA). A CORTECS® UPLC® C18 column (2.1 × 100 mm, 1.6 μm) was employed. The column temperature was set to 25 °C. The injection volume was 2 μL, the flow rate was 0.3 mL/min, and the total run time was 10 min. The mobile phases were 0.1% formic acid-aqueous solution (A) and methanol (B). The gradient elution program was as follows: 0–5 min, 95% A and 5% B; 5–6.5 min, 10% A and 90% B; 6.5–7 min, 40% A and 60% B; and 7–10 min, 95% A and 5% B. Mass spectrometric analysis was conducted using an electrospray ionization (ESI) source operated in positive-ion mode. The capillary voltage was set at 3.0 kV, the source temperature was 150 ℃, the cone gas flow was 150 L/h, and the desolvation gas flow was 600 L/h. Data were acquired in multiple reaction monitoring (MRM) mode.

Table S1. Methods for determining soil physicochemical properties

Physicochemical properties	Analytical method	Instrumentation
pH	Determination of soil pH by potentiometry (HJ 962–2018)	pH meter
Organic matter (OM)	Determination of soil organic matter and calculation of the carbon-to-nitrogen ratio (LY/T 1237–1999)	Analytical balance
Organic carbon (OC)	Determination of soil organic matter and calculation of the carbon-to-nitrogen ratio (LY/T 1237–1999)	Analytical balance
Total nitrogen (TN)	Determination of soil nitrogen: Kjeldahl method (Section 3.1, LY/T 1228–2015)	Analytical balance
Total phosphorus (TP)	Determination of soil phosphorus: acid-digestion method (Section 3.2, LY/T 1232–2015)	UV–visible spectrophotometer
Total potassium (TK)	Determination of soil potassium: acid-digestion method (Section 3.2, LY/T 1234–2015)	Inductively coupled plasma–optical emission spectrometer (ICP–OES)
Available phosphorus (AP)	Soil testing—Part 7: Determination of available phosphorus in neutral and calcareous soils (pH ≥ 6.5; Section 5.4, NY/T 1121.7–2014)	UV–visible spectrophotometer
Available potassium (AK)	Determination of soil available and slowly available potassium: determination of available potassium (Section 3.1, NY/T 889–2014)	Flame photometer
Soil water content (SWC) (%)	Determination of soil dry matter and moisture content by the gravimetric method: analysis of fresh soil samples (Section 7.2, HJ 613–2011)	Analytical balance
Ammonium nitrogen (NH_4^+-N)	Determination of soil nitrogen: indophenol blue colorimetric method (Section 6.1, LY/T 1228–2015)	Analytical balance
Nitrate nitrogen (NO_3^--N)	Determination of soil nitrogen: phenoldisulfonic acid colorimetric method (Section 5.1, LY/T 1228–2015)	UV–visible spectrophotometer

Table S2. Information on the analytical standards of bioactive compounds

Compound Name	CAS No.	Molecular Formula	MW	Purity (%)
3-Hydroxyflavone	577-85-5	C15H10O3	238.23	⩾98%
7-Hydroxyflavone	6665-86-7	C15H10O3	238.238	≥98%
Apigenin	520-36-5	C15H10O5	270.24	≥98%
Apigenin-7-O- (2G-rhaMnosyl) gentiobioside	174284-20-9	C33H40O19	740.66	99.9
Apigenin-7-O-β-D-glucoside	578-74-5	C21H20O10	432.38	98.4
Astragalin	480-10-4	C21H20O11	448.38	⩾98%
Astragaloside II	84676-89-1	C43H70O15	827.01	99.9
Astragaloside III	84687-42-3	C41H68O14	784.97	99.7
Astragaloside IV	84687-43-4	C41H68O14	784.97	99.5
Astragaloside I	84680-75-1	C45H72O16	869.05	99.2
Brachyoside B	86764-12-7	C36H60O10	652.86	99.3
Calycosin	20575-57-9	C16H12O5	284.26	≥98%
Calycosin-7-O-beta-D-glucoside	20633-67-4	C22H22O10	446.4	≥98%
Daidzein	486-66-8	C15H10O4	254.24	97.0
Formononetin	485-72-3	C16H12O4	268.26	98.7
Hyperoside	482-36-0	C21H20O12	464.38	≥98%
Isoastragaloside IV	136033-55-1	C41H68O14	784.97	99.9
Isoastragaloside I	84676-88-0	C45H72O16	869.05	99.7
Isoastragaloside II	86764-11-6	C43H70O15	827.01	99.1
Isorhamnetin	480-19-3	C16H12O7	316.26	≥98%
Kaempferol 3-O-robinobioside	17297-56-2	C27H30O15	594.52	99.9
Liquiritigenin	578-86-9	C15H12O4	256.25	97.7
Luteolin	491-70-3	C15H10O6	286.24	99.2
Mangiferin	4773-96-0	C19H18O11	422.34	≥98%
Narcissoside	604-80-8	C28H32O16	624.54	≥98%
Naringin	10236-47-2	C27H32O14	580.53	≥98%
Ononin	486-62-4	C22H22O9	430.4	≥98%
Orientin	28608-75-5	C21H20O11	448.38	98.8
Quercitrin	522-12-3	C21H20O11	448.38	98.0
Rutin	153-18-4	C27H30O16	610.52	≥98%
Salvigenin	19103-54-9	C18H16O6	328.32	99.9
Soyasaponin II	55319-36-3	C47H76O17	913.09	99.4
Soyasaponin I	51330-27-9	C48H78O18	943.12	99.4
Vitexin	3681-93-4	C21H20O10	432.38	99.9
Wogonin	632-85-9	C16H12O5	284.26	≥98%

Table S3. Calibration equations for the quantitative determination of flavonoid and saponin constituents in pure solvents

Compound Name	Regression equation	R^2	Range (ng/mL)
Isoastragaloside IV	y=552.979x+3911.48	0.9992	20-10000
Astragaloside III	y=101.644x-5788.13	0.9921	50-25000
Isoastragaloside II	y=42.7737x-3041.31	0.9905	50-25000
Isoastragaloside I	y=132.61x+13.8483	0.9933	20-25000
Liquiritigenin	y=1519.92x+33428.8	0.9925	20-5000
Luteolin	y=304.502x+2143.91	0.9921	20-1000
Ononin	y=2607.94x-20916.7	0.9901	20-25000
Calycosin-7-O-beta-D-glucoside	y=214.498x+4389.69	0.9948	20-25000
Brachyoside B	y=30.7656x+1436.49	0.9925	20-25000
Astragaloside IV	y=36.1544x+4672.94	0.9936	50-10000
Soyasaponin II	y=6.61837x+156.419	0.9907	20-10000
Soyasaponin I	y=160.023x-1323.29	0.9909	20-10000
Astragaloside I	y=60.0872x+3887.05	0.9930	50-25000
Astragaloside II	y=18.526x+85.6397	0.9952	50-10000
Calycosin	y=15.6631x-90.7314	0.9927	20-10000
Formononetin	y=3293.5x-17288.9	0.9924	20-1000

Table S4. The contents of 16 active compounds in the roots of 1- to 4-year-old *A. mongholicus* (mg/kg)

Compound Name	1YR	2YR	3YR	4YR
Isoastragaloside IV	5.44±0.35a	4.04±0.21c	4.94±0.14b	3.66±0.11d
Astragaloside III	87.80±4.62a	38.74±1.42b	35.22±0.62c	34.76±1.54c
Isoastragaloside II	23.40±2.18b	13.81±0.51d	27.38±0.92a	21.27±0.63c
Isoastragaloside I	32.40±2.02b	16.94±0.40c	37.98±2.11a	18.04±1.25c
Liquiritigenin	4.04±2.06a	4.42±1.30a	2.95±0.64a	3.09±0.53a
Luteolin	6.10±0.81a	4.43±0.44c	5.26±0.60b	4.02±0.25c
Ononin	203.53±77.55a	31.26±6.79b	64.58±10.00b	34.93±2.04b
Calycosin-7-O-beta-D-glucoside	809.20±54.64a	138.18±23.42c	354.27±36.82b	151.62±24.14c
Brachyoside B	7.29±0.71c	8.83±1.21bc	9.88±1.37b	13.87±2.25a
Astragaloside IV	40.98±2.67ab	34.08±1.43b	42.41±10.40a	38.68±6.57ab
Soyasaponin II	1.76±0.81b	2.76±1.29b	4.34±0.77a	2.02±0.53b
Soyasaponin I	77.41±4.89a	73.31±3.74a	38.23±11.21c	59.78±7.98b
Astragaloside I	462.20±55.82a	229.34±29.45b	411.87±76.93a	234.75±22.54b
Astragaloside II	68.50±5.14b	89.24±7.99a	90.52±9.60a	60.84±4.27b
Calycosin	84.01±13.22a	46.91±13.70b	70.15±13.63a	41.41±1.76b
Formononetin	14.57±3.11a	8.81±2.66b	12.48±4.01a	6.75±0.55b

The data in the table are the mean ± SD (n= 6).

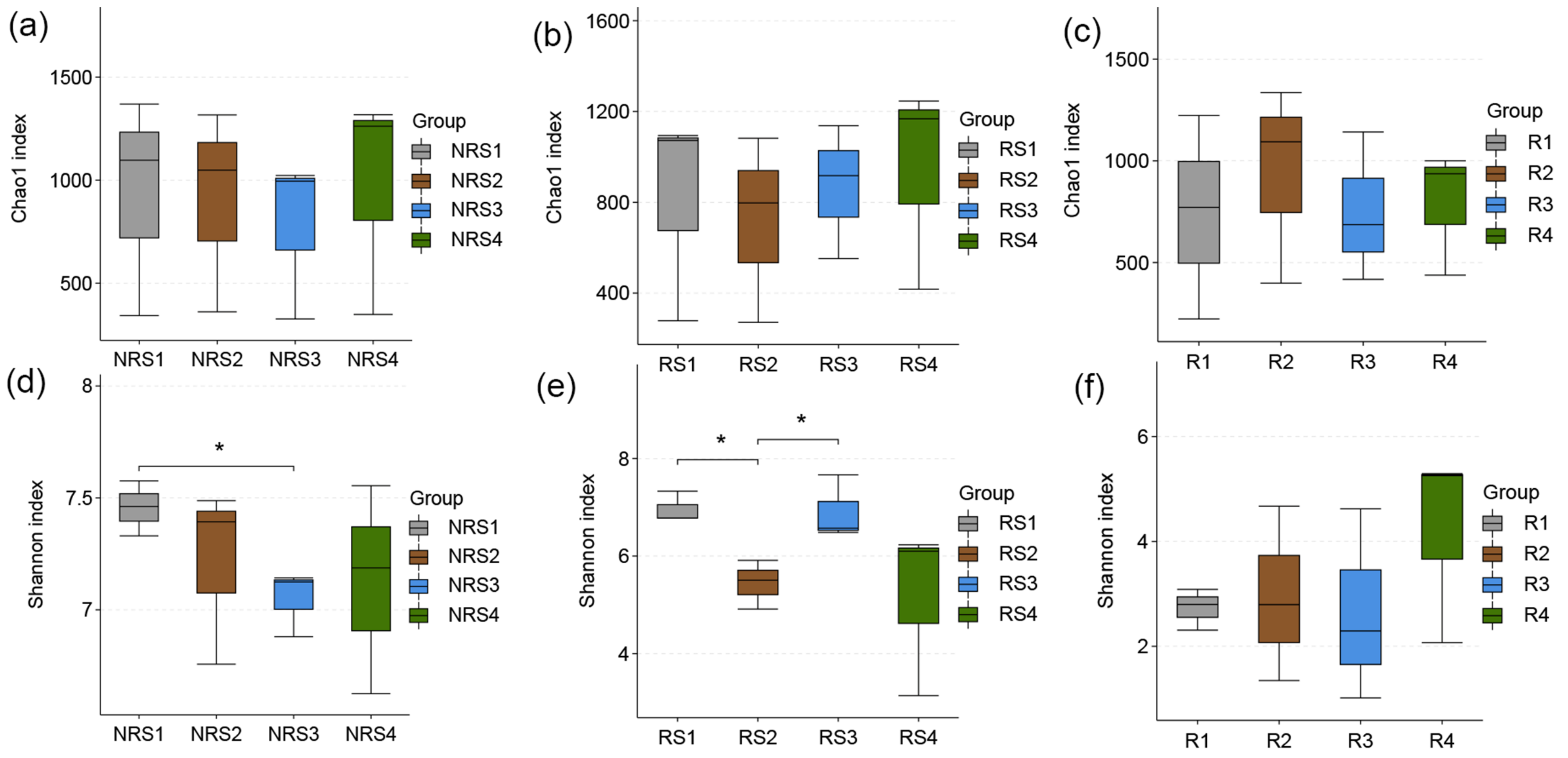

Figure S1. Analysis of α-diversity (Chao 1 and Shannon index) of fungal communities in non-rhizosphere soil (NRS), rhizosphere soil (RS), and root (R) of *A. mongholicus* from different cultivation years. (a) Bulk soil Chao 1 index; (b) Rhizosphere soil Chao 1 index; (c) Root Chao 1 index; (d) Bulk soil Shannon index; (e) Rhizosphere soil Shannon index; (f) Root Shannon index.

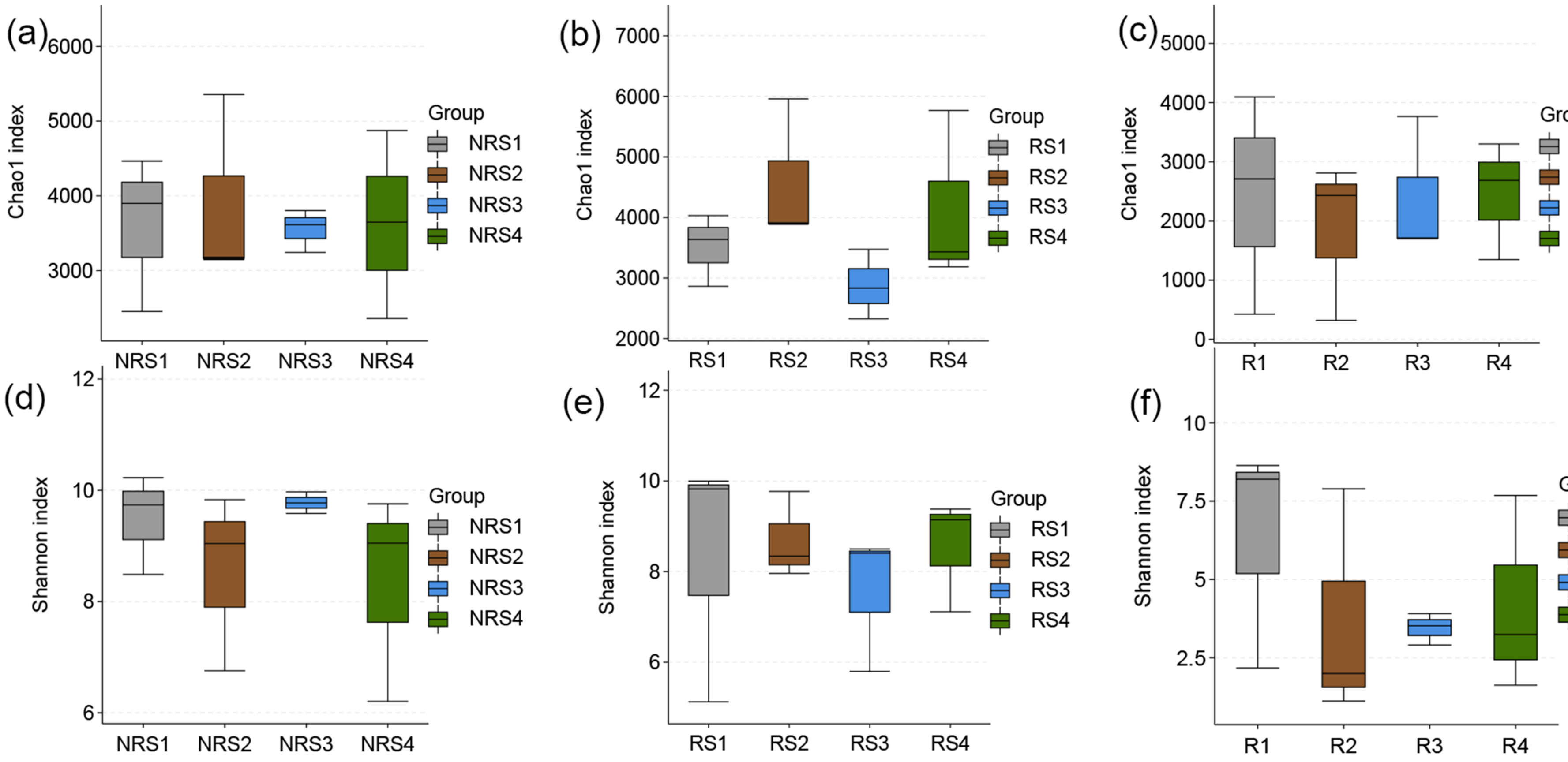

Figure S2. Analysis of α-diversity (Chao 1 and Shannon index) of bacterial communities in non-rhizosphere soil (NRS), rhizosphere soil (RS), and root (R) of *A. mongholicus* from different cultivation years. (a) Bulk soil Chao 1 index; (b) Rhizosphere soil Chao 1 index; (c) Root Chao 1 index; (d) Bulk soil Shannon index; (e) Rhizosphere soil Shannon index; (f) Root Shannon index.

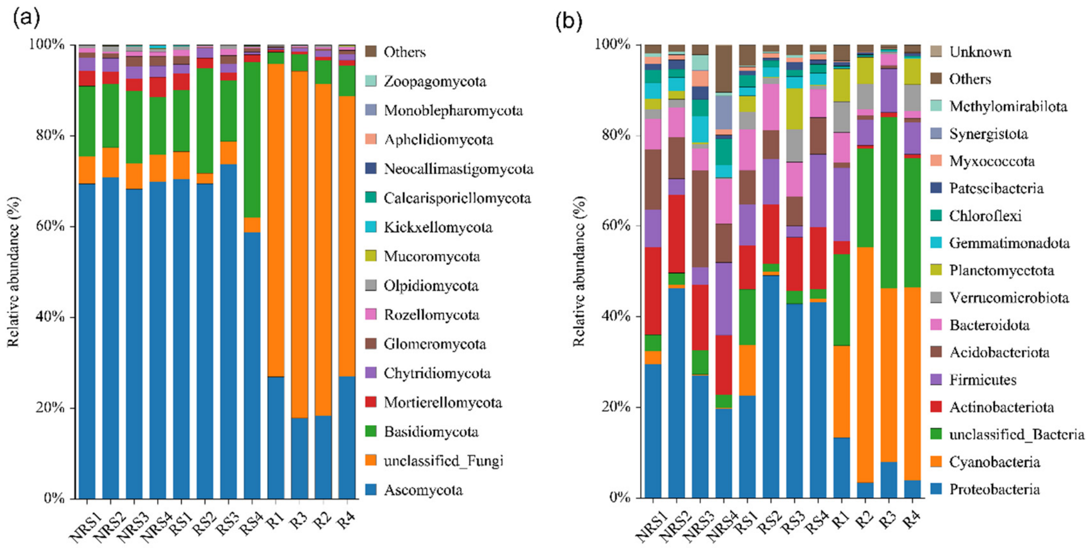

Figure S3. Phylum-level species composition and relative abundance of fungal (a) and bacterial (b) communities.

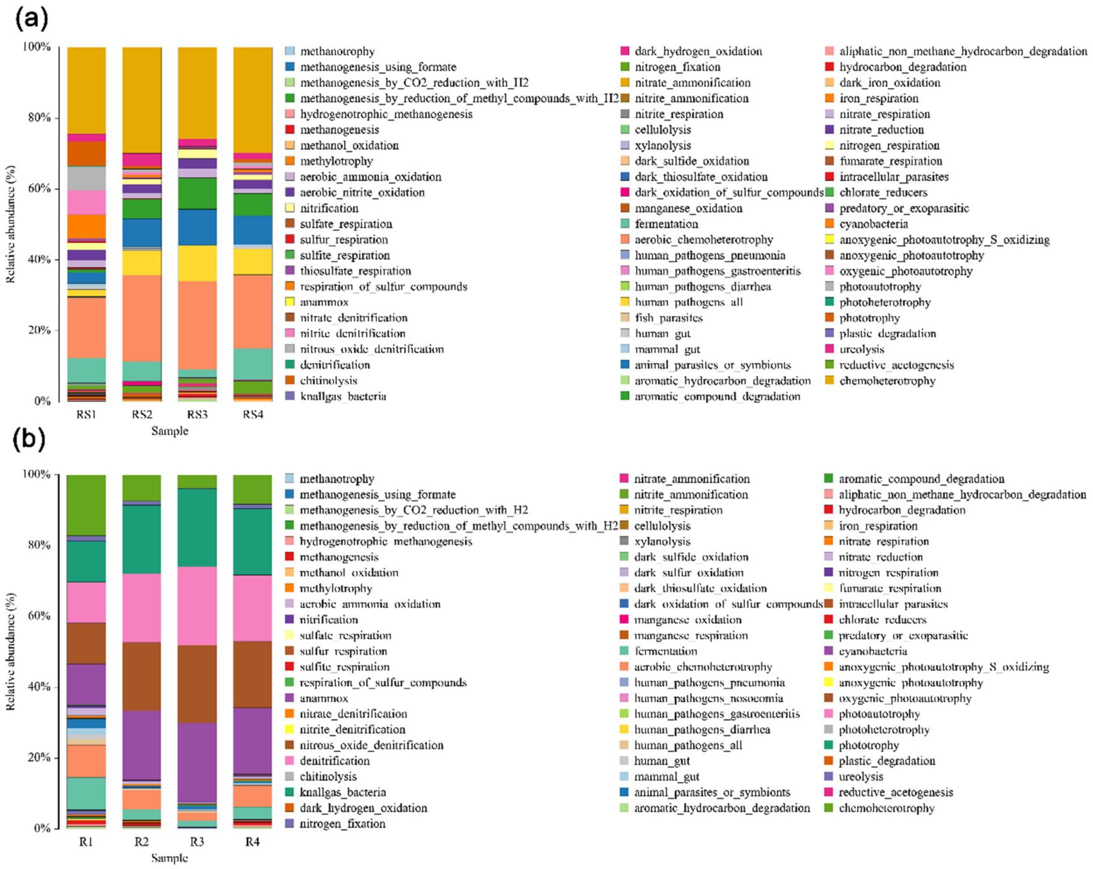

Figure S4. FAPROTAX functional annotation based on bacterial communities in the rhizosphere soil and root of *A. mongholicus* from different cultivation years.

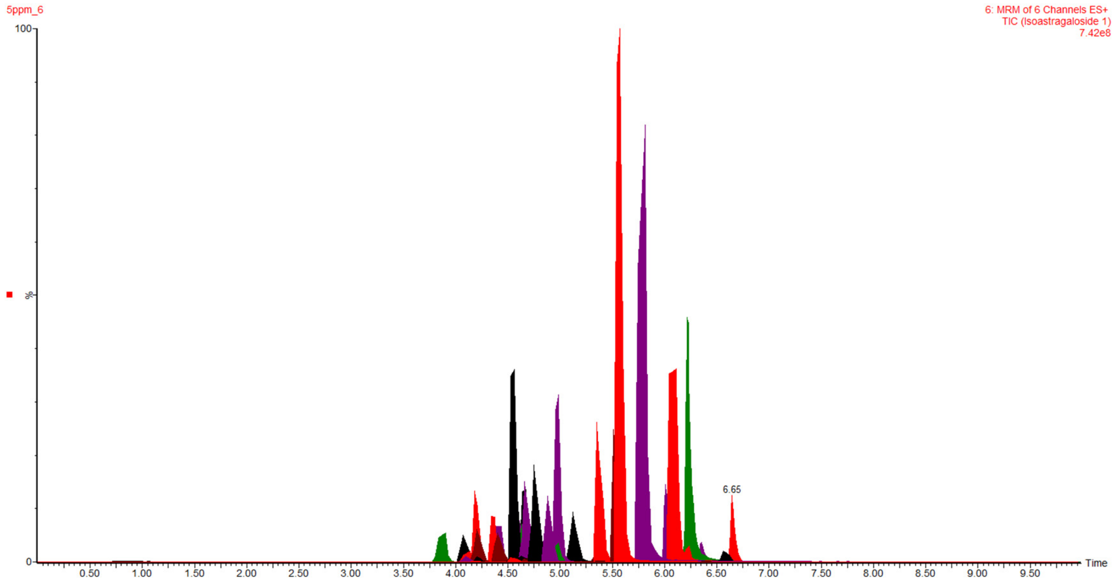

Figure S5. Total ion diagram of 35 active ingredients in standard solution.

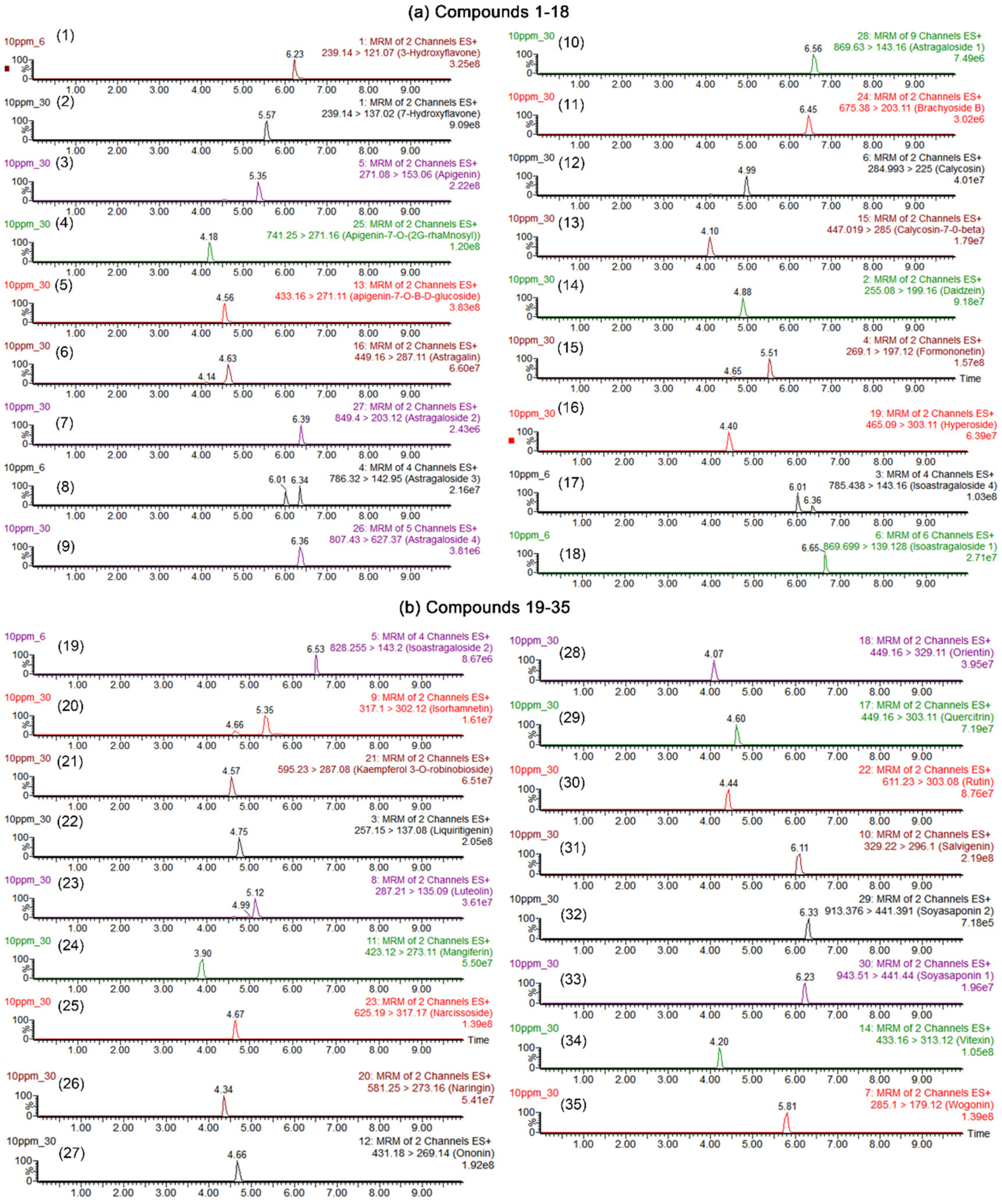

Figure S6. Quantifier ion chromatograms of 35 bioactive compounds. (1) 3-Hydroxyflavone; (2) 7-Hydroxyflavone; (3) Apigenin; (4) Apigenin-7-O-(2G-rhaMnosyl) gentiobioside; (5) Apigenin-7-O-β-D-glucoside; (6) Astragalin; (7) Astragaloside II; (8) Astragaloside III; (9) Astragaloside IV; (10) Astragaloside I; (11) Brachyoside B; (12) Calycosin; (13) Calycosin-7-O-beta-D-glucoside; (14) Daidzein; (15) Formononetin; (16) Hyperoside; (17) Isoastragaloside IV; (18) Isoastragaloside I; (19) Isoastragaloside II; (20) Isorhamnetin; (21) Kaempferol 3-O-robinobioside; (22) Liquiritigenin; (23) Luteolin; (24) Mangiferin; (25) Narcissoside; (26) Naringin; (27) Ononin; (28) Orientin; (29) Quercitrin; (30) Rutin; (31) Salvigenin; (32) Soyasaponin II; (33) Soyasaponin I; (34) Vitexin; (35) Wogonin.
